# Progress in Ophthalmology

**Published:** 1898-04-02

**Authors:** 


### Progress in Ophthalmology.

**Asepsis.**—Again the subject of asepsis against antisepsis has cropped up, and at the annual meeting of the British Medical Association, held at Montreal, a great many operating ophthalmic surgeons expressed themselves as greatly in favour of the former.[1] The large majority of those who spoke said that flushing out the conjunctival sac with boiled water, or at most a weak solution of boracic acid, was quite sufficient. All, with but one or two exceptions, were against any preliminary bandaging of the eye "to see how it would stand being bandaged," and considered that it was causing needless irritation, and should unhesitatingly be condemned. Mr. Noyes, who led the discussion, advocated the boiling of the instruments, including knives and needles, for five or ten minutes before use, and then placing them in a carbolic solution; while the President (Mr. Nettleship) said, in reference to the cutting instruments, such as knives and needles, that "dipping" them in boiling water was sufficient.

**Salicylic Acid Ointment in Spring Catarrh.**—Dr. Randolff,[2] at the same meeting, spoke of this treatment as making the prognosis in spring catarrh a much brighter one. He maintains that the ointment may be used as strong as six or eight grains to the ounce of lanoline, and that after the first few applications the patients stand it very well; and that it, to a large extent, relieved the itching and burning so common in this complaint.

**Conjunctivitis Preceding an Attack of Syphilitic Iritis.**—Mr. Juler,[3] in a lecture delivered before the Harvean Society on December 2nd, 1897, says, "A form of catarrhal conjunctivitis often occurs as a secondary manifestation preceding an attack of iritis. The ocular conjunctiva is much congested, and there is a muco-purulent discharge. It is owing to this catarrh that the iritis is sometimes neglected, for the one runs imperceptibly into the other; on the other hand, the redness of the eyeball may lead to the early use of atropine, and prevent an impending attack of iritis. The catarrh is usually symmetrical.

**Argentamine.**—Dr. Schulhof[4] says that Argentamine has been almost exclusively employed for several years in Professor Hoor's Clinic, in Klausenburg for affections of the conjunctiva, and that last year alone 240 cases were thus treated. He says that this remedy has proved superior to nitrate of silver. The conjunctiva treated with argentamine becomes paler in colour, the swelling is reduced, and the secretion decreased. Vascular contraction and astringent action are quite as apparent as with silver nitrate, while the patient does not complain of burning and pain, and can open the eyes without trouble immediately after the application. Argentamine is employed in 5 per cent. aqueous solution, being painted on or dropped into the eye once or twice daily, without subsequent washing with water or salt solution. Application three or four times daily is quite permissible, and gives rise to no danger of irritation. Even an irritated condition of the iris gives no contra-indication for its employment.

**Tattooing of Cornea.**—Fröhlich[5] considers the best results are obtained by using Von Hippel's corneal trephine of 3-5 mm. diameter, and that it should be used to a depth of one-third of a millimetre; the enclosed epithelial layer is then scraped away. The bare surface is scarified with a Graefe knife, and the thick paste of Indian ink rubbed on this prepared surface with back of a spoon. Finally, a light bandage is applied over the lids. He claims as an advantage of this method in cases of central corneal leucomata that the blackened area closely resembles a pupil in both shape and size, and that it is quickly covered with a transparent layer of epithelium, retaining a uniform blackness which does not decrease with time.

**Interstitial Keratitis.**—Mr. Juler,[6] in his second Harveian lecture, says of interstitial keratitis: "There are causes other than syphilis, especially tuberculosis. This, as a cause of interstitial keratitis is strongly upheld by Von Hippel, and his evidence is very convincing. For all that. we may positively assert that of all the cases of interstitial keratitis met with, two-thirds are due to hereditary syphilis." He draws attention to the lack of photophobia in interstitial keratitis, and says that this alone will help to distinguish it from superficial keratitis in which the photophobia is intense.

**Insertion of Artificial Globe into Tenon's Capsule.**—Morton, of New York, on the above subject, says[7] that in cases where the globe to be removed is either so lacerated or so atrophic as not to be able to contain a glass ball, as in Mule's operation, the following method may be found useful: The muscles are dissected up—after the conjunction is incised—to their insertion into the sclera, when they are then cut, the ends are tied by sutures, and the globe having been removed, the ends of the four recti are pulled by the sutures as far over the glass ball (which has been inserted into Tenon's capsule in the place of the eyeball) as possible, but the ends ought not to be fastened together. The inflammation which is set up holds and fastens these tendons before the sutures have ulcerated through, and the muscles then act on the glass sphere, and give it good movement. In the manipulation the oblique are cut away from the sclera and allowed to drop back into the orbit; the conjunctiva is sutured with fine Chinese silk, and in three days, he states, the socket will tolerate a glass eye being worn. He further says that there is very little irritation or chemosis, such as always follows a Mule's operation.

An interesting accident showing how an eye may be torn completely from its socket is reported in the

*British Medical Journal* of March 6th, 1898, by Mr. L. B. Todd; the patient in stepping from a dog-cart caught his eye on a blunt hook hanging from the ceiling; the muscles were torn clean away, and the eye was found lying on the cheek; the nerve torn off measured one and a half inches.

**Case of Upward Coloboma of the Iris with Subluxation of Lens Downwards.**—Dr. A. M. Gillivray[8] quotes a case which came under his care in which the patient, a man of 80, and suffering from cataract, had a coloboma of the iris and choroid upwards; the lens was extracted without much trouble, and patient made a rapid and uninterrupted recovery. He says from the development standpoint the case is of more than ordinary interest, as the position of the coloboma must be in direct antagonism to the theory that all colobomata of the iris are caused by a deficient closure of the fœtal cleft. The defect in this case is above while the fœtal cleft is below. The theory of impaired development at any part of the ciliary ridge would explain the coloboma in the zonule in this case, for if, during the process of development of the zonule, there is a gap in the ciliary body at any part of its circumference, it is impossible for the zonule to be formed opposite the gap.

[1] British Med. Journal, Jan. 8, 1893. [2] British Med. Journal, Jan. 8, 1898. [3] Lancet, Dec. 18, 1897. [4] The Therapist, Oct. 15, 1897. [5] British Med. Journal, Nov. 6, 1897. [6] British Med. Journal, Jan. 8, 1898. [7] Brit. Med. Journal, Jan. 8, 1898. [8] Brit. Med. Journal, Mar. 27, 1898.

